# Surgery on the aortic arch and feasibility of electroencephalography (SAFE) monitoring in neonates: protocol for a prospective observational cohort study

**DOI:** 10.1136/bmjopen-2025-106423

**Published:** 2025-07-10

**Authors:** William M McDevitt, Timothy J Jones, Laura Quinn, Christina L Easter, Jin Jing, M Brandon Westover, Barnaby R Scholefield, Stefano Seri, Nigel E Drury

**Affiliations:** 1 Neurophysiology, Birmingham Children’s Hospital NHS Foundation Trust, Birmingham, UK; 2 Cardiovascular Sciences, University of Birmingham, Birmingham, UK; 3 Paediatric Cardiac Surgery, Birmingham Children’s Hospital NHS Foundation Trust, Birmingham, UK; 4 Department of Applied Health Sciences, University of Birmingham, Birmingham, UK; 5 Department of Neurology, Harvard Medical School, Boston, Massachusetts, USA; 6 Department of Neurology, Beth Israel Deaconess Medical Center, Boston, Massachusetts, USA; 7 Pediatric Critical Care Medicine, Hospital for Sick Children, Toronto, Ontario, Canada; 8 Department of Pediatrics, University of Toronto, Toronto, Ontario, Canada; 9 Institute of Health and Neurodevelopment, Aston University, Birmingham, UK

**Keywords:** Electroencephalography, Paediatric cardiac surgery, Neonatology, Brain Injuries, Neurophysiology

## Abstract

**Abstract:**

**Introduction:**

While survival rates following neonatal surgery for congenital heart disease (CHD) have improved over the years, neurodevelopmental delays are still highly prevalent in these patients. After correcting for the CHD subtype, the severity of developmental impairment is dependent on multiple factors, including intraoperative brain injury, which is more frequent and more severe in those undergoing aortic arch repair with deep hypothermic circulatory arrest (DHCA). It is proposed that brain injury may be reduced if cooling is stopped at the point of electrocerebral inactivity (ECI) on electroencephalogram (EEG), but there is limited evidence to support this as few centres perform perioperative EEG routinely. This study aims to assess the feasibility of EEG monitoring during neonatal aortic arch repair and investigate the relationship between temperature and EEG to inform the design of a future clinical trial.

**Methods and analysis:**

Single-centre prospective observational cohort study in a UK specialist children’s hospital, aiming to recruit 74 neonates (≤4 weeks corrected age) undergoing aortic arch repair with DHCA. EEG will be acquired at least 1–3 hours before surgery, and brain activity will be monitored continuously until 24 hours following admission to intensive care. Demographic, clinical, surgical and outcome variables will be collected. Feasibility will be measured by the number of patients recruited, data collection procedures, technically successful EEG recordings and adverse events. The main outcomes are the temperature at which ECI is achieved and its duration, EEG patterns at key perioperative steps and neurodevelopmental outcomes at 24 months postsurgery.

**Ethics and dissemination:**

The study was approved by the Yorkshire and The Humber Sheffield National Health Service Research Ethics Committee (20/YH/0192) on 18 June 2020. Written informed consent will be obtained from the participant’s parent/guardian prior to surgery. Findings will be disseminated to the academic community through peer-reviewed publications and presentations at conferences. Parents/guardians will be informed of the results through a newsletter in conjunction with local charities.

**STRENGTHS AND LIMITATIONS OF THIS STUDY**

- This observational cohort study will evaluate the feasibility of a perioperative electroencephalogram (EEG) monitoring protocol for neonates undergoing aortic arch repair.
- It will assess the time and temperature required to achieve electrocerebral inactivity (ECI) during active systemic cooling in neonates undergoing deep hypothermic circulatory arrest.
- ECI could represent a personalised therapeutic target for the adequacy of cooling for brain protection prior to deep hypothermic circulatory arrest.
- Patient and public involvement has been a key component in the development, conduct and planned dissemination of this study since its inception.
- Data capture may be limited by the reliance on multiple individuals performing a relatively new task accurately, as any errors or inconsistencies in execution can result in incomplete or unusable data.

## Introduction

### Congenital heart disease (CHD) and brain injury

 In the UK, 13 babies per day are born with CHD, and a third of these will require surgery or other intervention during the first year of life.^1^ Neurological deficits and neurodevelopmental delay are among the most common extracardiac morbidities following surgery for CHD in infants^2 3^ and are more common and severe in those undergoing aortic arch repair.^4^

The reasons for this are complex and multifactorial. Impaired cerebral blood flow and autoregulation, reduced oxygen delivery, congenital brain abnormalities and acquired brain injury soon after birth can occur and may be exacerbated by prolonged cyanosis and hypoperfusion.^5^ During aortic arch repair, the infant is cooled to deep hypothermia before a period of circulatory arrest and has altered cerebral blood flow, both of which are risk factors for newly acquired brain injury.^6^ Postoperatively, the immature brain is more susceptible to seizures and stroke due to low cerebral blood oxygen saturations, hypotension and impaired cerebral autoregulation.^7^ These interrelated, cumulative and synergistic risk factors culminate in adverse neurodevelopmental outcomes that persist into later life.^8^

The recent James Lind Alliance Priority Setting Partnership in CHD identified improving organ protection during heart surgery and the effects on brain development and behavioural outcomes as key national research priorities in children with CHD.^9^ Despite current perioperative neuroprotective strategies, up to 44% of infants who undergo surgery for CHD develop newly acquired brain injury.^4^ Therefore, novel interventions to detect, monitor and limit perioperative brain injury may positively impact management and short- and long-term outcomes.

### Perioperative neuromonitoring using EEG

EEG may have a role in detecting, monitoring and limiting perioperative brain injury.^10 11^ EEG measures summated postsynaptic electrical potentials recorded from electrodes placed on the scalp.^12^ It is a sensitive and non-invasive method to assess real-time neurological function and is available at the bedside. In addition, continuous EEG (cEEG) recording may allow for the uninterrupted detection of potentially reversible pathology over long periods of time.^13^

Preoperative EEG monitoring detects pre-existing brain injury that occurs in patients with CHD due to physiological derangements, as well as genetic, epigenetic and developmental factors that alter cerebral perfusion and oxygenation.^14^ This is not a rare occurrence, as preoperative EEG abnormalities have been identified in 44%–60% of infants with CHD and are associated with abnormal brain development, structural injury, poor neurodevelopmental outcomes and increased risk of death.^15^,^18^ It is therefore even more important to minimise the risk of additional brain injury related to surgical repair in these vulnerable children and achieve the best long-term outcomes.

Intraoperative EEG may be used to guide the degree of hypothermia required before circulatory arrest,^19^ using EEG activity as a proxy measure of cerebral metabolism.^20^ As hypothermia progresses, EEG activity decreases in amplitude until no activity is recorded (electrocerebral inactivity (ECI)).^21^ As the aim of hypothermia is to reduce cerebral metabolic demand, ECI is proposed as the point at which cerebral metabolism is low enough to best tolerate the ischaemic nature of circulatory arrest^22^ and achieving ECI prior to arrest is therefore neuroprotective.

However, in a recent literature review on the role of EEG during surgery with hypothermic circulatory arrest (HCA), we found that while EEG has been used to guide cerebral protection, there is a lack of comparative data to demonstrate the benefit of EEG monitoring.^10^ This was due to variability between clinical outcome metrics used; EEG acquisition, recording and interpretation parameters; ECI definitions; the temperature at which ECI occurred and protocols for performing EEG-guided HCA.

Postoperative EEG can be used to detect and direct treatment for subclinical seizures that occur in up to 20% of infants following surgery for CHD.^23^ The immature brain is more susceptible to seizures in response to cerebral ischaemia or haemorrhage, and cerebral autoregulation is impaired perioperatively, making these infants more vulnerable to seizures. In those who develop seizures, up to 85% are subclinical,^24^ and seizure burden is associated with postoperative outcome.^25^ For this reason, guidelines recommend postoperative cEEG monitoring in neonates undergoing CHD surgery with cardiopulmonary bypass (CPB).^26^

Although indications for perioperative EEG monitoring may appear clear, it is only used by 14%–17% of European adult aortic centres intraoperatively,^27^ and 32%, 16% and 20% of neonatal cardiac surgery centres use preoperative, intraoperative and postoperative amplitude-integrated EEG monitoring, respectively.^28^ Postoperatively, 96% of North American paediatric intensive care units can perform cEEG monitoring,^29^ but few use it routinely for neonates with CHD.^30^ This variability in practice highlights the heterogeneous nature of EEG monitoring, which may be due to practical barriers or the feasibility of recording perioperative EEG in critically ill neonates undergoing cardiac surgery.

### Rationale

In this study, we explore the feasibility of EEG monitoring in neonates undergoing aortic arch repair with deep hypothermic circulatory arrest (DHCA) and assess whether EEG monitoring could be used to optimise perioperative neuroprotection. As the feasibility of a perioperative neuromonitoring protocol in this setting is largely unknown, we will assess recruitment capability, data collection procedures and the rate of technically successful recordings. The main outcomes are whether ECI is achieved before DHCA and the temperature at which it occurs, the duration of ECI and EEG patterns associated with late neurodevelopmental outcomes. A better understanding of these outcome measures will inform the design of a future clinical trial evaluating the role of EEG monitoring during neonatal aortic arch surgery.

## Methods and analysis

### Design

Surgery on the aortic arch and feasibility of EEG (SAFE) monitoring is a single-centre prospective observational cohort study on the feasibility and role of perioperative EEG monitoring during paediatric cardiac surgery. The full study protocol is available as online supplemental appendix A.

#### Inclusion and exclusion criteria

Neonates (≤4 weeks corrected age) undergoing aortic arch repair, either as an isolated procedure or as part of a more complex repair (eg, Norwood, arterial switch) using CPB and DHCA with occlusion of the head and neck arteries at Birmingham Children’s Hospital (BCH) will be included. Potential participants will only be excluded if the parent or guardian is unable or unwilling to provide informed consent.

Initially, during the feasibility phase, parents of infants ≤2 years of age were approached to take part. Following feedback during the external funding review and moving into the full observational cohort study, inclusion criteria were revised to only include neonates ≤4 weeks corrected age.

#### Recruitment

Monthly reports of mid-trimester antenatal ultrasound scans confirming the presence of CHD within the West Midlands, UK, will be reviewed by the principal investigator (PI). Soon after birth, these patients are admitted to the cardiac ward or paediatric intensive care unit (PICU). Additional cases present in a typical manner (tachypnoea, tachycardia, feeding difficulties, lower limb arterial pulse and pressure abnormalities) that result in hospital admission and echocardiogram confirming hypoplasia of the aortic arch. The PI will screen admissions to the cardiac ward and PICU twice a day and review biweekly joint cardiac conferences to identify eligible patients.

**Figure 1:**
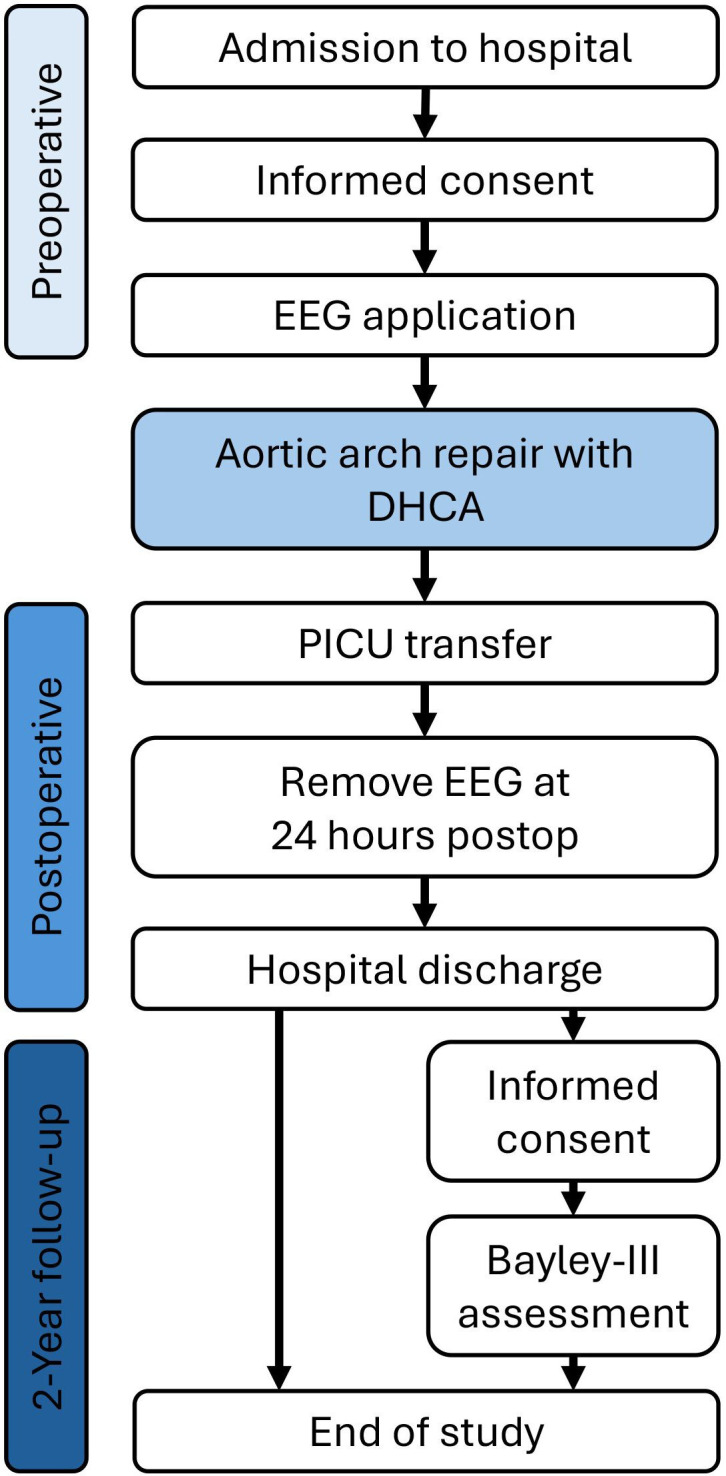
Flow diagram for the surgery on the aortic arch and feasibility of EEG monitoring study. DHCA, deep hypothermic circulatory arrest; EEG, electroencephalography; PICU, paediatric intensive care unit.

The parent or guardian of potential participants will be approached during their child’s inpatient admission, before CHD surgery. They will be given a parent information sheet (online supplemental appendix B) and given at least 12 hours to consider their child’s participation and ask questions. Written informed consent (online supplemental appendix C) will be obtained by the PI or another member of the research team prior to enrolment. The participant pathway through the study is shown in figure 1. If the parent or guardian cannot understand written or spoken English, the BCH interpreting and cultural insight department, or BCH staff members who speak their native language, will be approached to provide interpretation. If unavailable, a remote interpreting service app (LanguageLine Solutions) will be used. Parents or guardians who also give consent to be contacted for long-term follow-up will be reapproached approximately 2 years (22–26 months) following their child’s surgery to attend a neurodevelopmental outcome assessment clinic.

### Routine procedures

#### Perioperative management

Neonates will receive intravenous prostaglandin infusion to maintain patency of the ductus arteriosus as needed. Critically unwell neonates may be ventilated and sedated, predominantly with morphine. Clonidine or milrinone may be prescribed to maintain adequate preoperative blood pressure. Intraoperatively, inhaled and intravenous agents will be used for anaesthesia and analgesia, with fluorinated hydrocarbons preferred to propofol for anaesthetic induction and maintenance. Adjuncts to alter haemodynamics will be used at the discretion of the anaesthetist and perfusionist. Intraoperative phenylephrine and isoflurane will be favoured for vasoconstriction and dilation, respectively. Invasive arterial and central venous pressure will be monitored continuously along with heart rate, pulse oximetry, cerebral tissue oxygen saturation via near-infrared spectroscopy (NIRS) and other routinely recorded perioperative data in line with current recommendations.^31^ Postoperative sedation and analgesia will be maintained via intravenous opioids or opioid-sparing analgesia where appropriate.

#### Surgical and postoperative care

Repair of the aortic arch will be performed in line with best clinical practice. After transfer to the operating room, the surgical checklist will be completed, the patient prepped and draped, and the chest opened through a median sternotomy. Following systemic anticoagulation with heparin, CPB will be achieved via arterial cannulation of the innominate artery through a polytetrafluoroethylene tube or via the patent ductus arteriosus. The venous cannula will typically be placed within the right atrium. During CPB, mean arterial pressure will be kept between 30 and 50 mm Hg and central venous pressure around 0 mm Hg.

Progressive hypothermia for end-organ protection will follow at a rate not faster than 1°C per minute and for a total duration of at least 20 min prior to starting DHCA. An aortic cross-clamp will be applied to the proximal ascending aorta with intermittent antegrade cold cardioplegia given via the aortic root for myocardial protection. In patients with a hypoplastic ascending aorta, cardioplegia will be administered via the arterial inflow cannula following circulatory arrest and occlusion of the descending aorta and head and neck vessels. The aortic arch will be repaired by resection of hypoplastic tissue and end-to-end primary anastomosis, with or without left subclavian artery overlay and/or augmentation using a pulmonary homograft patch. Concomitant pulmonary artery banding and associated intracardiac malformation repair (eg, ventricular septal defect closure) will be performed, as required. If aortic arch repair is performed as part of Norwood (stage 1) palliation for hypoplastic left heart syndrome, a shunt will be placed to either the left or right pulmonary artery to provide pulmonary blood flow, and an atrial septal defect (ASD) will be created if not already present.^32^

DHCA will be used in all patients to facilitate exposure and opening of the aortic arch as well as creating an ASD in single ventricle circulations. During the repair of the aortic arch, selective antegrade cerebral perfusion (SACP) will be used via the innominate artery with control of the proximal head and neck vessels and right atrial venous drainage. SACP will be commenced at 50% of the calculated patient cardiac output, and the cerebral perfusion pressure will be monitored via an arterial cannula in the right arm. Upon completion of the arch repair, the arch will be deaired and secured, and all clamps on head and neck vessels will be removed, followed by full flow CPB and systemic rewarming to 36°C. During rewarming, the arterial perfusate temperature will be kept within 10°C of the patient’s temperature and will not exceed 37°C.

Following rewarming, CPB will be weaned and discontinued, and anticoagulation reversed with protamine. In the event of difficulty separating from CPB or marked haemodynamic instability, subjective and objective measures of ventricular function will be obtained, and inotropic support will be instituted at the discretion of the operating team. Once haemodynamic stability and haemostasis have been achieved, the chest will be closed at the discretion of the surgical team, and the patient will be transferred to the PICU. Standard postoperative care will proceed with the transfer to the ward once routine PICU discharge criteria have been met. All decisions regarding the escalation of therapy will be made by the clinical team responsible for the care of the child.

### Study-related procedures

#### Electroencephalography

All participants will have up to 23 scalp electrodes applied according to published guidelines.^33 34^ A collodion-based adhesive and micropore tape will be used to secure electrodes to the scalp. Frontal electrodes will be placed equidistant between FP1 and F3 and Fp2 and F4 so that NIRS probes can be secured to the forehead for routine perioperative cerebral oximetry (NIRS) monitoring. EEG will be acquired via an ambulatory recording device (Morpheus Home LTM or SD LTM 64 Express, Micromed UK) synchronised with an Intellivue X^3^ monitor (Philips, Farnborough, UK), which records vital signs, NIRS and core body temperature data. All data will be coded with a unique study number and stored electronically on secure IT infrastructure. Core body temperature will be measured via nasopharyngeal and oesophageal probes.

EEG recordings will start 1–3 hours prior to the anticipated time of surgery or the night before. Recording will continue intraoperatively, blinded to the clinical team and will not influence surgical, perfusion or anaesthetic management. Postoperatively, the patient will be transferred to PICU where the ambulatory EEG recording will be stopped and downloaded. EEG acquisition will continue for 24 hours on a dedicated cerebral function analysing monitor (NatusNeuroWorks, Guildford, UK) within the PICU. EEG data that could inform postoperative care (eg, identification of subclinical seizures or patterns observed in hypoxic ischaemic encephalopathy) will be shared with the clinical team.

#### Neurodevelopmental assessment

The parents or guardians of participants in whom consent for future contact is obtained will be reapproached approximately 2 years (22–26 months) following surgery for a single assessment of neurodevelopment. With additional consent, participants will be assessed by a trained physiotherapist using the third edition of the Bayley Scales of Infant and Toddler Development (Bayley-III)^35^ during a 90-min outpatient appointment. Bayley-III is validated for the evaluation of developmental functioning in children between 1 and 42 months of age.^36^ It will be used to assess function in five developmental domains (cognition, language, motor, socioemotional and adaptive behaviour), and results will be compared with a standardised normative age-matched sample. Attempts will be made to schedule the assessment alongside pre-existing hospital appointments, and results will be shared with the child’s clinical team.

#### Clinical data collection

Demographics, surgical and medical variables, and adverse events before, during and after surgery will be collected using a patient case report form (CRF). Bayley-III record forms and questionnaires, and hard copies of CRFs will be stored in a research master site file.

### Outcome measures and follow-up

Study outcomes, measurements and assessment time periods are summarised in tables1  2). The feasibility of perioperative EEG monitoring will be determined by the participant recruitment rate, data collection procedures, technically successful EEG recordings and adverse events.

**Table 1:** Surgery on the aortic arch and feasibility of EEG monitoring data collection and measurement methods

Data	Measurement (primary source)	Time period
Feasibility	Rate of recruitment, adverse events, complete data sets and technically successful recordings (case report form)	Continuous
Demographic	Date of birth, gestational and corrected age, height and weight (medical notes)	Baseline
Clinical information	Diagnosis, preoperative resting oxygen saturations, comorbidities, primary surgical procedure, preoperative medication, blood lactate on PICU admission, postoperative medications, duration of PICU stay and hospital stay, date of death, seizures, clinical examination, brain injury metrics identified via clinical observation and neuroimaging (medical notes)	Continuous
Brain activity	Continuity, symmetry, synchrony, voltage, variability, reactivity, dysmaturity, encephalopathy grade, seizure burden; duration and morphology of cortical waveforms (EEG)	Preoperative, intraoperative and postoperative
Surgical information	Intraoperative drugs, cardiopulmonary bypass, cross-clamp, deep hypothermic circulatory arrest, selective antegrade cerebral perfusion and cooling and rewarming duration and rates, vital signs (surgical notes).	Intraoperative
Temperature	Nasopharyngeal and oesophageal (Intellivue X^3^)	Intraoperative and postoperative
Cerebral oximetry	Cerebral blood oxygen saturation (near-infrared spectroscopy)	Intraoperative and postoperative
Neurodevelopment	Total raw score, scaled and composite scores with percentile rank and 95% CIs; developmental age and quality descriptors in five domains: cognition, language, motor, socioemotional and adaptive behaviour (Bayley-III)	24-month follow-up

CI, Confidence interval; EEG, electroencephalography; PICU, paediatric intensive care unit.

**Table 2:** Schedule of the timepoints data will be collected during the surgery on the aortic arch and feasibility of EEG monitoring study

Timepoint	Enrolment	Preoperative	Intraoperative	Postoperative	2-year follow-up
Eligibility screen	X				
Informed consent	X				
Demographic information		X			
EEG and Intellivue X^3^ synchronisation		X			
EEG application		X			
EEG recording		X	X	X	
Temperature ECI achieved*			X		
Cerebral oximetry recording			X	X	
Feasibility data				X	
Clinical information			X	X	
Surgical information			X	X	
EEG interpretation				X	
Bayley III neurodevelopmental assessment					X

* Temperature recorded continuously from the point nasopharyngeal and oesophageal probes are placed postanaesthetic induction and preinitiation of cardiopulmonary bypass, up until postsurgical removal while still under general anaesthesia.

ECI, Electrocerebral inactivity; EEG, electroencephalography.

#### Primary outcome

The proportion of participants who achieve ECI, defined as <2 μV EEG activity for a 3-min period, during cooling before the start of DHCA, and in these, the temperature (°C) and variability (±SD) at which ECI occurs.

#### Secondary outcomes

- The relationship between whether or not ECI is achieved during cooling before DHCA and clinical outcome measures.
- The duration of ECI (min) and its relationship with clinical outcome measures.
- The morphology, amplitude and continuity of EEG activity during hypothermia, DHCA, (SACP and rewarming to normothermia.
- Preoperative and postoperative background EEG patterns and seizure burden (absent, present and status epilepticus). This will be assessed retrospectively by specialist clinical neurophysiologists, with reference to published articles.^37^,^40^

All patients will be followed for 30 days or until hospital discharge, whichever is sooner, and at approximately 2-years postaortic arch repair for those who attend long-term follow-up. The Bayley-III assessment will be used to determine which children are delayed in comparison to standardised normative datasets.

### Analysis

#### Sample size

During the feasibility phase, 17/30 (57%) patients achieved ECI before DHCA at a mean temperature of 22.6°C (SD±2.9). We estimate that by recruiting a total of 74 participants, 41 will achieve ECI before DHCA with the same mean temperature at ECI with a 95% CI around the estimate of ±0.9°C. There is no robust data on the rate of secondary outcomes in neonatal aortic arch repair, so these will be considered exploratory.

#### Statistical analysis

Counts and percentages will be used to summarise categorical data and means with SD or medians with IQR used to summarise continuous variables, as appropriate. Grouped differences will be assessed using χ^2^ for categorical variables and a parametric alternative for continuous variables. Correlation coefficients will be used to investigate the relationship between variables, and linear regression will be performed to look for associations between the temperature at which ECI is achieved before DHCA and collected covariates (table 1). Logistic regression will be performed to look for associations between covariates and whether ECI is achieved before DHCA or not. In regression analyses, there will be adjustments for potential confounding factors, such as perioperative drug regimes, preoperative neurological abnormalities and CHD complexity, as these may affect clinical outcomes. Estimates and 95% CIs will be reported. Analysis will be performed using Stata, and p<0.05 will be considered significant.

#### EEG interpretation

Each EEG will be visually assessed by a clinical neurophysiologist blinded to participant outcome, and cortical electrical activity will be interpreted in the domains listed in table 1. Where appropriate, artefacts will be removed from EEG data epochs via independent component analysis, a data segmentation algorithm or spatial pattern filters.

**Figure 2:**
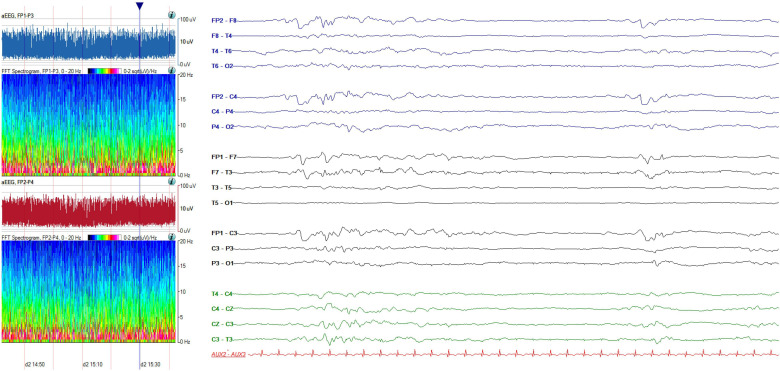
Left: Quantitative EEG. aEEG shows peak-to-peak EEG amplitude (y-axis, semilog scale) over time (x-axis) for left (blue) and red (right) cerebral hemispheres. Fast Fourier Transform spectrograms show EEG frequency (y-axis) over time (x-axis), with spectral power in colour (z-axis). All plots display 1 hour of data; red gridlines mark 10-min intervals. Right: EEG corresponding to the vertical blue line in aEEG/spectrograms. 12 seconds shown at 10 µV/mm sensitivity. aEEG, amplitude integrated EEG; EEG, electroencephalography.

Persyst software (Persyst, Solana Beach, California, USA) will be used to create colour density spectral arrays of EEG frequencies and power characteristics, and the peak-to-peak amplitude of EEG activity will be plotted and evaluated on semilogarithmic scales as a function of time, as shown in figure 2. This will be used primarily to enhance interpretation of EEG patterns listed in table 1.

For quantitative analysis, the EEG will be segmented into epochs in a semiautomated manner. The proportion of suppressed cortical activity in each epoch will be calculated and plotted over temperature with a regression line fitted to show the strength of the relationship between temperature and EEG suppression. The duration and amplitude of bursts at different core body temperatures and stages of surgery will be compared by calculating 95% CI of mean differences through bootstrap resampling and using cumulative distribution functions (CDF). Differences between CDFs will be compared using the two-sample Kolmogorov-Smirnov test. Power spectra will be computed at different temperatures and surgical stages. Significant differences will be identified using either the two group test for spectra^41^ or by calculating 95% CIs of mean differences through bootstrap resampling. Periods where CIs do not overlap will be considered significantly different. Quantitative analyses and EEG segmentation will be performed using Matlab (Mathworks, Natick, Massachusetts, USA), and p<0.05 will be considered significant.

### Safety reporting

Adverse events which may be directly related to the conduct of the study will be recorded, and serious adverse events reported to the study sponsor (Birmingham Women’s and Children’s NHS Foundation Trust) and the Yorkshire and The Humber Sheffield Research Ethics Committee. Adverse reactions to study interventions will be documented as file notes in the study site file. Participants in the study are undergoing open heart surgery, and therefore adverse events unrelated to study interventions are anticipated.

### Patient and public involvement (PPI)

PPI has been a central component in the development, conduct and planned reporting of this study since its inception. Parents of children who had previously undergone cardiac surgery were contacted through two local charities: Young at Heart and Droitwich DoubleBeat British Heart Foundation Fundraising group. Five parents formed the PPI group and through virtual meetings, reviewed and amended the parent information sheets, consent forms and lay summaries to enhance clarity and readability for a lay audience. Strategies to recruit from underserved patient groups were discussed, and recommendations were put into action. They also advised on the most important aspects of neurodevelopment to measure. The outcomes of the study will be communicated through individual parent feedback and a charity newsletter, both of which will be co-produced with the charities and parents.

## Ethics and dissemination

### Ethical approval

The study was approved by the Yorkshire and The Humber Sheffield Research Ethics Committee (20/YH/0192) on 18 June 2020 and the National Health Service (NHS) Health Research Authority (279319) on 3 July 2020. It is sponsored by Birmingham Women’s and Children’s NHS Foundation Trust (BCH/123). As described above, parents/guardians of participants provide written informed consent prior to enrolment in the study, including future contact for late neurodevelopmental assessment. The first patient was recruited on 1 September 2020, and recruitment is currently ongoing, with a planned completion date of 31 March 2026. This report is based on protocol V.5.0 dated 29 April 2025, which was approved on 9 June 2025.

### Protocol amendments

Since the original ethical approval, four amendments to the protocol have been sought and approved during study recruitment, with the following significant changes:

- With additional funding, the addition of the Bayley-III neurodevelopmental assessment performed at 2 years postsurgery (SA001, approved 18 August 2022). As this was implemented before the first participant reached 2 years of age, all surviving participants had the opportunity to undergo late assessment.
- Revised inclusion criteria from participants ≤2 years of age to ≤4 weeks corrected age, as recommended by external fellowship funding application reviewers, and extension of the study duration to enable late follow-up of all participants (SA002, approved 19 December 2023). This amendment will have a minor impact on the analysis, as those participants already recruited but beyond the neonatal period (>4 weeks corrected age, n=4) will be excluded.
- Export of pseudonymised study data outside of the European Economic Area to enable collaboration with the team in Boston, Massachusetts, USA (SA003, approved 13 March 2024).
- Protocol amendment to clarify the objectives, outcome measures and sample size calculation, resulting from moving from the feasibility phase to the full observational cohort study phase, in line with the NIHR funding award (SA004, approved 9 June 2025).

### Dissemination

The study findings will be submitted for presentation at national and international meetings, and manuscripts will be prepared for submission to leading peer-reviewed journals. The authorship of the main study outputs will include members of the study team and named collaborators. Anonymised individual participant data collected during the study will be available upon request, at the discretion of the study team, following publication of the study results.

Parents of the children participating in the study will receive a written lay summary of the results, co-produced with the PPI group. Young at Heart and Droitwich DoubleBeat will report key study findings in their local charity newsletters to reach a wider audience of those affected by CHD.

The first author is PI of the study and takes responsibility for the integrity of this protocol report, which adheres to the Strengthening the Reporting of Observational Studies in Epidemiology statement.^42^ All authors have read and agree to the manuscript as written.

## Supplementary material

10.1136/bmjopen-2025-106423 online supplemental file 1

10.1136/bmjopen-2025-106423 online supplemental file 2

10.1136/bmjopen-2025-106423 online supplemental file 3
