# Supplementary material for: Surgery on the aortic arch and feasibility of electroencephalography (SAFE) monitoring in neonates: protocol for a prospective observational cohort study
Source: BMJ Open. 2025 Jul 10;15(7):e106423. doi: 10.1136/bmjopen-2025-106423 (PMC12258354; doi:10.1136/bmjopen-2025-106423)
Supplement: online supplemental file 1 [file bmjopen-15-7-s001.pdf]

Protocol: Surgery on the Aortic arch and Feasibility of EEG monitoring

Protocol version: 5.0
Study phase: Observational cohort
Acronym: SAFE monitoring
Sponsor: Birmingham Women's and Children's NHS Foundation Trust, Steelhouse Lane, Birmingham B4 6NH.
Protocol date: 29/04/2025

Collaborators <Address and Names redacted>

<Address>: <Name> (Chief investigator), <Name> (Principal Investigator), <Name>, <Name>

<Address>: <Name>, <Name>

<Address>: <Name>, <Name>

<Address>: <Name>, <Name>, <Name>

<Address>: <Name>, <Name>, <Name>

<Address>: <Name>, <Name>

<Address>: <Name>, <Name>

Table of contents

1. Protocol summary
2. Background
    I. Introduction
    II. Rationale and objectives
    III. Potential benefits and risks
3. Recruitment
    I. Study population
    II. Inclusion and exclusion criteria
    III. Screening and participant enrolment
4. Procedures
    I. EEG
    II. Clinical data collection
5. Outcome measures
    I. Primary outcome
    II. Secondary outcome
6. Follow up
7. Sample size calculation
8. Statistical analysis
9. Ethical considerations
10. Data handling
    I. Data collection
    II. Data monitoring
    III. Data sharing
11. Reporting adverse events
12. Indemnity
13. Financial arrangements
14. Authorship and publication
15. Amendment history
16. Appendix: SAFE-Follow-up assessment of Infant Neurodevelopment (SAFE-FIN) Sub-study
17. References

1. Protocol summary

Title: Surgery on the aortic arch and feasibility of EEG (SAFE) monitoring

Population: Target population is all neonates (≤4 weeks corrected age) requiring aortic arch surgery at Birmingham Children's Hospital (BCH).

Study duration: Participants are enrolled at one time point and provide electroencephalography (EEG) data for analysis and use in future research projects. The study will finish September 2028.

Aim: To identify if EEG monitoring can be used to optimise neuroprotection perioperatively and therefore contribute to risk reduction. We will do this by tracking temperature-dependent changes in brain electrical activity during cooling to 18 degrees Celsius (the temperature used by BCH team) in neonates undergoing aortic arch repair, and identify the temperature at which electro-cerebral inactivity (ECI) is achieved. Pre- and Post-operative EEG will be recorded to identify groups which require increased perioperative resource.

Primary objective: To evaluate whether the temperature at which ECI on EEG is achieved is a modifiable risk factor associated with the long-term neurodevelopmental outcomes of neonates undergoing aortic arch surgery with deep hypothermic circulatory arrest (DHCA).

Secondary objectives: To report perioperative EEG patterns, seizure burden, and neurodevelopmental outcome in this previously unmonitored group.

Funding: HEE/NIHR Doctoral Clinical and Practitioner Academic Fellowship (NIHR302896) awarded to William McDevitt (2023-2026). The follow-up sub-study is funded by a research grant from Birmingham Women's and Children's Hospital Charity (37-6-206).

Schematic of study design: Patients scheduled for aortic arch repair will be identified from the surgical waiting list by the Principal Investigator (PI) and communicated to the research team via a secure messaging app (eg. WhatsApp). A Parent Information Sheet will be provided to the parent or guardian of potential study participants, and they will be asked to give written informed consent for their child to participate (Figure 1). Neurophysiology will complete a case report form (CRF), apply electrodes to the scalp, synchronize EEG to internal clock of intelliVue X3®, and acquire EEG. After surgery, EEG electrodes will be transferred to a paediatric intensive care unit (PICU) cerebral function analysing monitor (CFAM) for 24 hours of postoperative EEG acquisition. The Chief Investigator (CI) and PI will analyse EEG and temperature data. Routinely collected clinical and surgical variables will be recorded by the PI.

Follow-up: With consent, the parents or guardians of participants enrolled in the study will be re-approached 22-26 months following surgery to participate in the SAFE-Follow-up of Infant Neurodevelopment (SAFE-FIN) sub-study. With additional consent, neurodevelopmental outcome will be assessed by a trained physiotherapist using the Bayley-III scale of infant development (see Appendix).

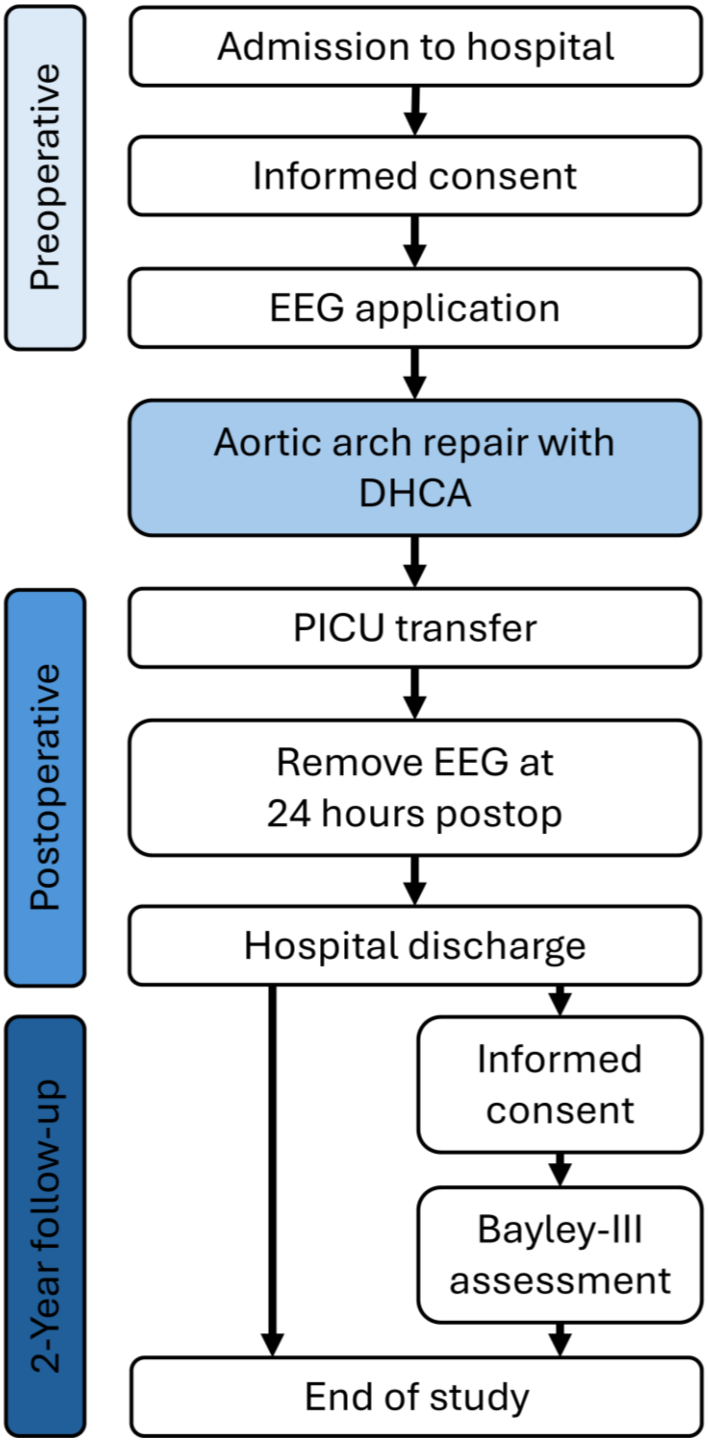

Figure 1: The flow of participants through the SAFE study.

2. Background

i. Introduction

Aortic arch repair is a complex operation which requires specialised neuroprotective strategies when arteries supplying the brain with blood are temporarily occluded during the repair. Reducing cerebral metabolism via hypothermia protects against the effects of cortical ischaemia and optimal cerebral protection is thought to occur during periods of electro-cerebral inactivity (ECI) as measured via EEG.[1]

ECI, defined as <2μV EEG activity for a 3-minute period, occurs at core temperatures between 28-12°C.[1] Sixty percent of adults achieve ECI at 18°C, the temperature used by the BCH team,[2] but recent data in infants suggests more variability.[3-4] Without EEG, multisite temperature monitoring is recommended to ensure adequate brain cooling (class 1 recommendation, level B evidence) but time to ECI, and cortical temperature, are not precisely predictable from oesophageal or nasopharyngeal temperature (temperature monitors used at BCH).

Up to 20% of potential study participants will develop sub-clinical seizures perioperatively,[5-7] and the only way to detect these is via EEG. Preoperative EEG may help identify which patients are more likely to require specialized neuroprotection as certain patterns will highlight central nervous system abnormalities. Although there is currently insufficient evidence to support routine EEG monitoring for the study population in question[1] it is feasible that:

- Preoperative EEG could identify patients at high risk of neurological complications.
- There may be inter-individual variation in the degree of hypothermia required to achieve ECI, and thereby optimal neuroprotection.
- Postoperatively, a substantial proportion of patients may develop subclinical seizures which go undetected in the absence of EEG monitoring and are associated with adverse neurological outcome.[8]

ii. Rationale & objectives

The addition of perioperative EEG for neonates undergoing aortic arch repair could ensure that:

- Those with abnormalities detected via EEG are identified preoperatively.
- Hypothermia is not excessive beyond the point of ECI, which has been reported to predict poor neurodevelopmental outcome.[4]
- Current practice is sufficient to provide optimal neuroprotection.
- Perioperative subclinical seizures are identified and treated in a timely manner.

Currently there is a lack of evidence in this field which this study will contribute to.

The objectives of the study are to:

1. Evaluate whether the temperature at which ECI on EEG is achieved is a modifiable risk factor associated with the long-term neurodevelopmental outcomes of neonates undergoing aortic arch surgery with deep hypothermic circulatory arrest (DHCA).
2. Report perioperative EEG patterns, seizure burden, and neurodevelopmental outcome in this previously unmonitored group.

iii. Potential benefits and risks

Known potential benefits

EEG interpretation may have clinical significance to the participant's care. Seizures, epileptiform activity, immaturity, evidence of hypoxic ischaemic encephalopathy (HIE), and Bayley-III scores will be disclosed to the clinical team looking after the participant. By improving our understanding of optimal neuroprotection during surgery, this study has the potential to improve the outcomes of future patients undergoing aortic arch repair.

Known potential risks

The procedures involved in the study are routine, involve minor risks only and do not alter patient management. The risk of EEG includes skin abrasions and mild discomfort during electrode application, which will be minimised by ensuring only qualified staff apply electrodes.

3. Recruitment

i. Study population

Neonates (≤4 weeks corrected age) undergoing surgical repair of the aortic arch with deep hypothermic circulatory arrest (DACA), either as an isolated procedure or as part of a more complex congenital cardiac procedure, eg. Norwood operation for hypoplastic left heart syndrome.

ii. Inclusion and exclusion criteria

Inclusion criteria: Neonates (≤4 weeks corrected age) undergoing aortic arch repair using cardiopulmonary bypass and DHCA, with occlusion of the head and neck arteries.

Exclusion criteria: Parent or guardian is unable or unwilling to provide informed consent.

iii. Screening and participant enrolment

Participants will be identified from the surgical waiting list by the PI and communicated to the research team via a secure messaging app (e.g. WhatsApp) in which key study collaborators will be included. The PI will know when the potential participant is due to come in (TCI) for the procedure and the date and time appropriate to seek informed consent. A Parent Information Sheet will be provided to the parent or guardian of potential study participants at least 12 hours before consent is sought. The PI will seek informed consent from the parent or guardian of study participants prior to surgery.

The parent or guardian is free to withdraw consent at any time without providing a reason. If at any time the parent or guardian request withdrawal, the data collected up until that time may still be included in the analysis. The PI will document study withdrawal in the medical notes.

4. Procedures

i. EEG

All participants will have scalp electrodes applied in accordance with published guidelines,[9-10] consisting of up to 23 electrodes, with a minimum of 15 (Figure 2). A collodion adhesive and micropore tape will secure electrodes to the scalp. Frontal electrodes will be placed equidistant between FP1/Fp2 and F3/F4 if near infrared spectroscopy (NIRS) is used to monitor cerebral tissue oxygen saturation. EEG will be acquired via an ambulatory recording device [Morpheus Home LTM or SD LTM 64 Express, Micromed] and data will be coded with a unique study number and stored electronically on a secure part of the NHS Trust IT network. Temperature will be measured via a nasopharyngeal temperature probe and recorded via intellivue X^3 monitors.

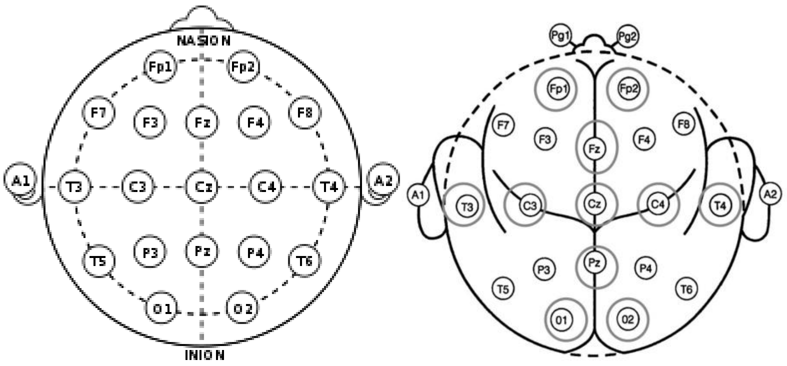

Figure 2. Left: The international 10-20 system for EEG electrode placement. Right: a modified version for neonates. NB. A1 and A2 will not be applied. In addition to the diagrams, ground and reference electrodes will be placed in an "electrically neutral" location on the scalp; 2 ECG electrodes will be applied to the torso.

ii. Clinical data collection

Participants' CRF will be entered onto a secure electronic database within Birmingham Children's Hospital (BCH). Offline/hard copies of CRFs will be stored within a research master site file located within the neurophysiology department of BCH. The CRF will be populated by the PI. Other routinely collected clinical variables will be obtained from the cardiac database. Research collaborators from the Neurophysiology department will assess pre- and postoperative EEG and seizure burden in accordance with previously published guidelines.[11-12]

5. Outcomes measures

i. Primary outcome

The proportion of participants who achieve ECI during cooling before the start of DHCA, and in these, the temperature (degrees Celsius) and variability (±SD) at which ECI is achieved.

ii. Secondary outcomes

- The relationship between whether or not ECI is achieved during cooling before DHCA and clinical outcome measures.
- The duration of ECI (minutes) and its relationship with clinical outcome measures.
- The morphology, amplitude and continuity of EEG activity during hypothermia, DHCA, selective antegrade cerebral perfusion (SACP) and rewarming to normothermia.
- Pre- and postoperative background EEG patterns and seizure burden (absent, present, status epilepticus). This will be assessed retrospectively by specialist clinical neurophysiologists, with reference to published articles.[11-13]

6. Follow-up

All patients will be followed for 30 days or until hospital discharge, whichever is sooner. A follow-up sub-study will be conducted at approximately 2 years following surgery, to identify whether perioperative EEG activity is associated with long-term neurodevelopmental outcome. With consent, the parents or guardians of participants will be re-approached at 22-26 months following surgery to participate in the SAFE-Follow-up of Infant Neurodevelopment (SAFE-FIN) sub-study. With additional consent, neurodevelopmental outcome will be assessed by a trained physiotherapist using the Bayley-III scale of infant development. Additional information on this sub-study is provided in the Appendix.

7. Sample size calculation

During the feasibility phase, 17/30 (56%) patients achieved ECI before DHCA at a mean temperature of 22.6°C (SD ±2.9). We estimate that by recruiting a total of 74 participants, 41 will achieve ECI before DHCA with the same mean temperature at ECI with a 95% confidence interval around the estimate of ±0.9°C. There is no robust data on the rate of the secondary outcomes in neonatal aortic arch repair so these will be considered exploratory.

8. Statistical analysis

Baseline and procedural characteristics will be summarized using median with interquartile ranges, or mean with standard deviation for continuous variables, and counts with percentages for categorical variables. Study feasibility will be measured by counts and percentages of successful patient recruitment,

data collection procedures and technically successful EEG recordings. The temperature (degrees Celsius) and variability (±SD) at which ECI is achieved and evolution of continuity will inform EEG utility in cardiac surgery. Secondary analysis will compare demographic, clinical and EEG variables between those with ECI at DHCA and those without to identify subgroups which may require increased neurocritical care resource.

9. Ethical considerations

Ethical approval will be sought via the integrated research application system (IRAS), NHS Research Ethics Committee (REC), health research authority (HRA) and the research and development office at BCH. The analysis of the EEG may have clinical significance. Seizures, epileptiform activity, immaturity, and evidence of HIE will be disclosed to the participant's clinical team.

10. Data handling

i. Data collection

The CRF and cardiac database are the primary data collection instruments for the study. All data requested must be recorded and missing data must be explained. The CRF or emailed data sheets can be printed off in paper form but must be kept in the master site file. Access to intellivue X^3 data will be made available to the PI via the PICU study collaborators.

ii. Data monitoring

Data from CRFs and the cardiac database will be inputted into an electronic database and stored within a secure network at BCH which will be maintained by the PI. Data entered will be checked for missing or unusual values via range checks. If any problems are identified the PI will inform the CI and resolve the issue.

The original signed consent form will be stored in the master site file and copies given to the parent or guardian and placed in the medical notes. Individual participant medical information obtained as a result of this study is considered confidential. CRFs and EEG data will be identified by unique study identifiers only.

iii. Data sharing

In the event of collaborations with centres, both within and outside of the United Kingdom, pseudonymised EEG data will be uploaded to either the BCH SharePoint, a cloud-based platform that requires two-factor authentication for access, or a BCH administered, password protected external hard drive. SharePoint data in transit will be protected using best-in-class-encryption. The pseudonymisation key will not be shared with collaborators, so the data will be shared anonymously. All pseudonymised data downloaded to non-BCH computers will be deleted after use. After analysis, EEG data, including interpretation, will be uploaded onto the BCH SharePoint and the BCH administered, password protected secure external hard drive. Only the PI will know the password for the BCH administered external hard drive. A data sharing agreement will be signed by each centre we collaborate with.

We also plan to share anonymised EEG and associated clinical data with the Brain Data Science Platform (https://bdsp.io/about/), an international data repository for neurophysiological, brain imaging, genetics, omics, and associated clinical data and open-source software, which is run by the Clinical Data Animation Center and the McCance Center for Brain Health, Boston, MA.

11.Reporting adverse events

The PI will record adverse reactions (AR) which may be directly related to the conduct of the study and document them in the participant's medical notes. ARs will be disclosed to the local research ethics committee providing ethical approval in an annual report. The participant's consultant will also be informed.

The PI and CI will assess AR causality as:

- Possibly: Temporal relationship of the onset of the event, relative to administration of the intervention, is reasonable but the event could have been due to another, equally likely cause.
- Probably: Temporal relationship of the onset of the event, relative to the administration of the intervention, is reasonable and the event is more likely explained by the intervention than any other cause.
- Definitely: Temporal relationship of the onset, relative to administration of the intervention, is reasonable and there is no other cause to explain the event, or a re-challenge (if feasible) is positive.

12.Indemnity

BCH research and development office insurance will apply as appropriate.

13.Financial arrangements

This main study is funded by an HEE/NIHR Doctoral Clinical and Practitioner Academic Fellowship (NIHR302896). The follow-up sub-study is funded by a research grant from Birmingham Women's and Children's Hospital Charity (37-6-206).

14.Authorship and publication

The PI and CI, Nigel Drury, Tim Jones and Barney Scholefield will form the basis of the publication committee and advise on drafting and submitting manuscripts following the advice of the entire research team.

15.Amendment history

Amendment number	Date of amendment	Protocol version	Summary of amendment
SA001	25/07/2022	v2.0	- Addition of SAFE:FIN sub-study, with funding<br>- Extension of recruitment period, with funding
SA002	08/11/2023	v3.0	- Inclusion criteria revised to only neonates<br>- Extension to study duration to complete recruitment and 2-year follow-up sub-study<br>- Correction of typographical errors
SA003	21/02/2024	v4.0	- Added section on data sharing with collaborators
SA004	29/04/2025	v5.0	- Revision to study phase, moving from feasibility to observational cohort study. Objectives, outcome measures, and sample size revised accordingly.

Appendix: SAFE-Follow-up assessment of Infant Neurodevelopment (SAFE-FIN) sub-study

Population: All neonates (≤4 weeks corrected age) recruited to the SAFE Monitoring study at BCH in whom consent for future contact for determining development was obtained.

Study duration: With consent, the parents or guardians of participants will be re-approached at 22-26 months following surgery for a single assessment of neurodevelopment.

Aims: To 1) identify whether perioperative EEG activity is associated with long-term neurodevelopmental outcome, 2) assess feasibility of conducting follow-up neurodevelopmental assessments in this population.

Primary objective: Assess neurodevelopmental outcome following aortic arch surgery in neonates approximately 24 months post procedure using the Bayley-III scale of infant development.

Secondary objectives: Evaluate whether the duration of electrocerebral inactivity (ECI), or not achieving ECI prior to deep hypothermic circulatory arrest (DHCA); seizure burden and other EEG patterns are associated with Bayley-III scores. We will also determine whether late follow-up neurodevelopmental assessments are feasible in this population.

Schematic of study design: Participant flow through the sub-study is shown in figure 3. The PI will inform physiotherapy when neonatal SAFE Monitoring study patients are approaching the 24-month post procedure time point. The PI will agree several dates and times when the assessment could take place with physiotherapy and neurophysiology. The PI will then contact the families of recruited neonates and ask whether they would be interested in attending a follow-up appointment to assess their child's development. Where possible, these will be arranged in-line with BCH outpatient clinic appointments to reduce the burden on the family. The PI will confirm the appointment date and time with the parent or guardian and inform physiotherapy. All assessments will take place in the department of neurophysiology. The parent or guardian has already given written informed consent to be contacted about a follow-up assessment of their child's development. Physiotherapy will perform the Bayley-III assessment and document results on a dedicated score sheet. After the assessment, the physiotherapy team will email the PI the results of the assessment and provide the PI with the original hard copy for the site file. Bayley-III results will be shared with the child's consultant Paediatrician.

Procedures: All participants will be assessed using the third edition of the Bayley Scales of Infant and Toddler Development (Bayley-III) by a trained physiotherapist.[14] This scale is an individually administered instrument that assesses developmental functioning of children between 1 month and 42 months of age. It is used to identify suspected delay in children consistent with current academia pertaining to child development. It can also assist paediatricians with intervention planning and other important clinical services. Additionally, the Bayley-III is designed to promote an understanding of the child's strengths and weaknesses in relation to five developmental domains: cognitive, language, motor, social-emotional, and adaptive behaviour. We anticipate the assessment appointment will last approximately 90 minutes, with an additional 90 minutes for analysis and interpretation of findings after the appointment.

Primary outcome: Adaptive behaviour score with percentile rank.

Secondary outcomes: The scaled and standard score, including percentile rank and age-equivalent for Language, Cognitive, Motor, and Social-Emotional Bayley-III domains. Number of participants recruited to follow-up assessment.

Analysis: Scores for each domain and subtest will be summarized using mean and standard deviation.

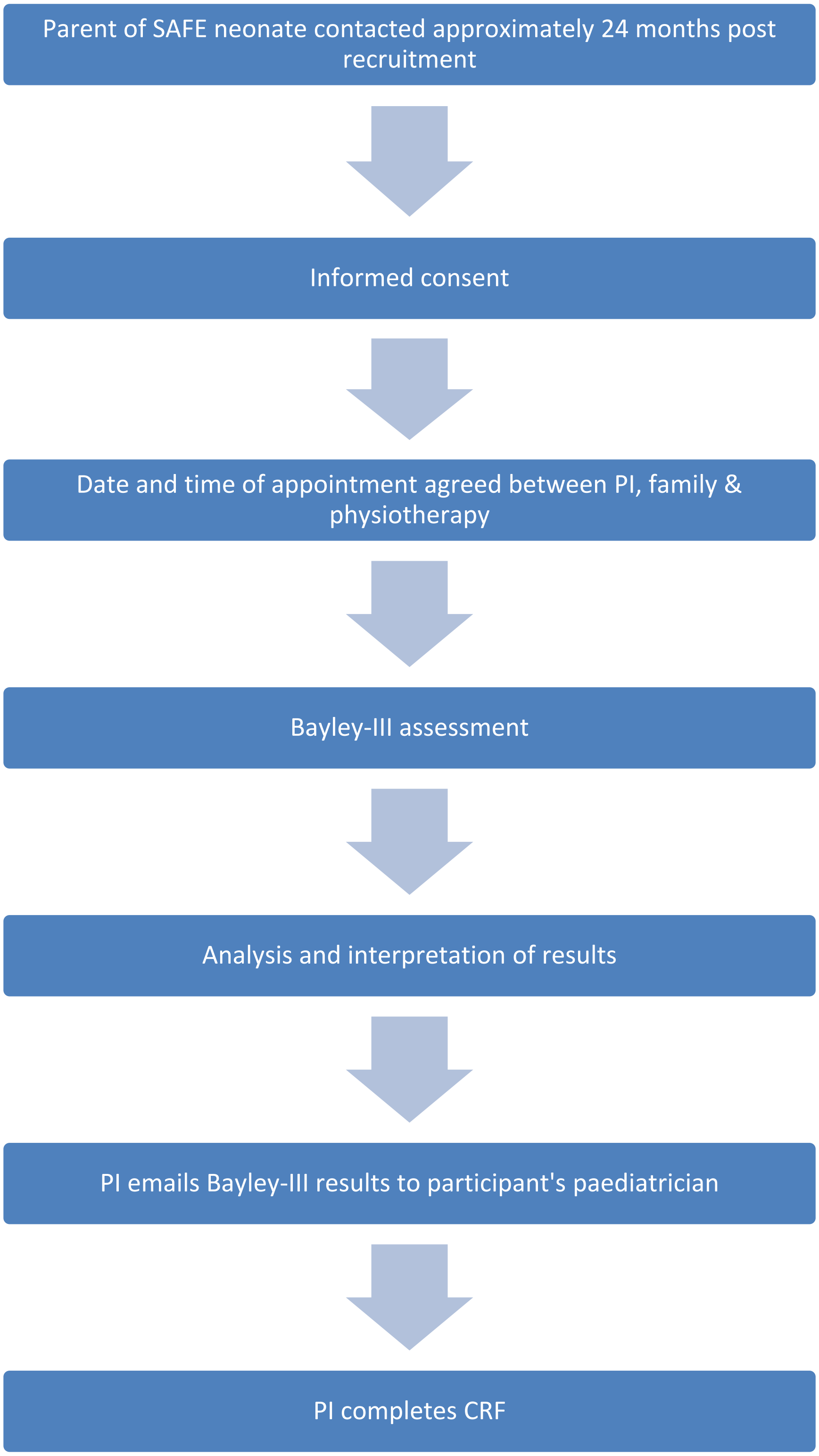

Figure 3: Participant flow through the sub-study

16.References

1. Czerny M ,Schmidli J et al. Current options and recommendations for the treatment of thoracic aortic pathologies involving the aortic arch: an expert consensus document of the European Association for Cardio-Thoracic surgery (EACTS) and the European Society for Vascular Surgery (ESVS). *European Journal of Cardio-Thoracic Surgery.* 2019; **55**(1): 133-162.

2. Stecker MM, Cheung AT, Pochettino A, Kent GP, Patterson T, Weiss SJ et al. Deep hypothermic circulatory arrest: I. Effects of cooling on electroencephalogram and evoked potentials. *Ann Thorac Surg.*2001;**71**:14–21.

3. Mavroudis CD, Mensah-Brown KG, Ko TS, Boorady TW, Massey SL, Abend NS, Nicolson SC, Morgan RW, Mascio CE, Gaynor JW, Kilbaugh TJ. Electroencephalographic response to deep hypothermic circulatory arrest in neonatal swine and humans. *The Annals of thoracic surgery*. 2018; **106**(6):1841-6.

4. Seltzer L, Swartz MF, Kwon J, Burchfiel J, Cholette JM, Wang H, Sweeney D, Adams HR, Meagher C, Angona R, Guillet R. Neurodevelopmental outcomes after neonatal cardiac surgery: role of cortical isoelectric activity. *The Journal of thoracic and cardiovascular surgery*. 2016; **151**(4):1137-44.

5. Helmers SL, Wypij D, Constantinou JE, et al. Perioperative electroencephalographic seizures in infants undergoing repair of complex congenital cardiac defects. *Electroencephalogr Clin Neurophysiol* 1997; 102: 27–36

6. Naim MY, Gaynor JW, Chen J, Nicolson SC, Fuller S, Spray TL, Dlugos DJ, Clancy RR, Costa LV, Licht DJ, Xiao R. Subclinical seizures identified by postoperative electroencephalographic monitoring are common after neonatal cardiac surgery. The Journal of thoracic and cardiovascular surgery. 2015 Jul 1;150(1):169-80

7. Gaynor JW, Nicolson SC, Jarvik GP, Wernovsky G, Montenegro LM, Burnham NB, Hartman DM, Louie A, Spray TL, Clancy RR. Increasing duration of deep hypothermic circulatory arrest is associated with an increased incidence of postoperative electroencephalographic seizures. *The Journal of thoracic and cardiovascular surgery.* 2005; **130**(5):1278-86.

8. Herman ST, Abend NS, Bleck TP, Chapman KE, Drislane FW, Emerson RG, Gerard EE, Hahn CD, Husain AM, Kaplan PW, LaRoche SM. Consensus statement on continuous EEG in critically ill adults and children, part I: indications. Journal of clinical neurophysiology. 2015; **32**(2):87.

9. Shellhaas RA, Chang T, Tsuchida T, Scher MS, Riviello JJ, Abend NS, Nguyen S, Wusthoff CJ, Clancy RR. The American Clinical Neurophysiology Society's guideline on continuous electroencephalography monitoring in neonates. *Journal of clinical neurophysiology*. 2011;**28**(6):611-7.

10. Kuratani J, Pearl PL, Sullivan LR, Riel-Romero RM, Cheek J, Stecker MM, Orta DS, Selioutski O, Sinha SR, Drislane FW, Tsuchida TN. American clinical neurophysiology society guideline 5: minimum technical standards for pediatric electroencephalography. *The Neurodiagnostic Journal*. 2016;**56**(4):266-75.

11. Hirsch LJ, LaRoche SM, Gaspard N, Gerard E, Svoronos A, Herman ST, Mani R, Arif H, Jette N, Minazad Y, Kerrigan JF. American clinical neurophysiology society's standardized critical care EEG terminology: 2012 version. *Journal of clinical neurophysiology*. 2013; **30**(1):1-27.

12. Tsuchida TN, Wusthoff CJ, Shellhaas RA, Abend NS, Hahn CD, Sullivan JE, Nguyen S, Weinstein S, Scher MS, Riviello JJ, Clancy RR. American clinical neurophysiology society standardized EEG terminology and categorization for the description of continuous EEG monitoring in neonates: report of the American Clinical Neurophysiology Society critical care monitoring committee. *Journal of Clinical Neurophysiology*. 2013; **30**(2):161-73.

13. Pressler RM, Boylan GB, Morton M, Binnie CD, Rennie JM. Early serial EEG in hypoxic ischaemic encephalopathy. *Clinical neurophysiology*. 2001; **112**(1):31-7.

14. Bayley N. Bayley scales of infant and toddler development: Bayley-III. Harcourt Assessment, Psych. Corporation; 2006.
