# Supplementary material for: Surgery on the aortic arch and feasibility of electroencephalography (SAFE) monitoring in neonates: protocol for a prospective observational cohort study
Source: BMJ Open. 2025 Jul 10;15(7):e106423. doi: 10.1136/bmjopen-2025-106423 (PMC12258354; doi:10.1136/bmjopen-2025-106423)
Supplement: online supplemental file 2 [file bmjopen-15-7-s002.pdf]

Birmingham Women's and Children's
NHS Foundation Trust

PARENT/GUARDIAN INFORMATION SHEET

Surgery on the Aortic arch and Feasibility of EEG Monitoring (SAFE Monitoring).

Chief Investigator: Prof. Stefano Seri, Consultant in Clinical Neurophysiology, BCH.

An invitation to participate in research: We want to give your child the opportunity to participate in a research study conducted by the heart surgery and neurophysiology research teams at Birmingham Children's Hospital.

Before you decide, you need to understand why the research is being done and what it would involve for you and your child. Please read the following information carefully and take time to decide whether you would like your child to take part. If there is anything that is not clear or you would like more information please ask the Principal investigator, Will McDevitt (contact details below).

Why is my child being invited to take part?

Your child has been referred to the Heart Surgery team for an operation which includes repair of the aorta, the main blood vessel coming out of the heart. We are approaching all parents whose baby requires aortic arch surgery to take part in this study and aim to recruit at least 74 babies over a 6-year period.

Why is the study being done?

We want to see if monitoring the brain with electroencephalography (EEG) can be used to optimise brain protection during, and around the time of aortic arch surgery. EEG works by placing electrodes on the scalp and measuring brainwaves (please refer to *figure one* for image).

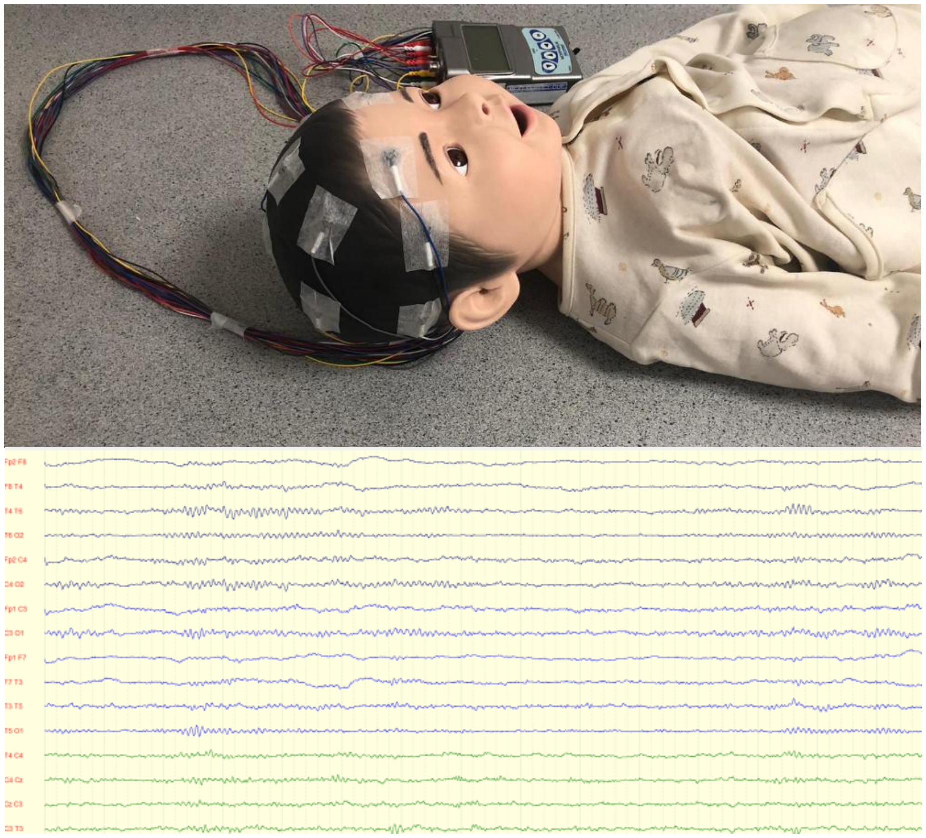

Figure one: This is where the EEG electrodes will be placed on your child (top picture) and the type of activity we will be recording (bottom picture).

The amount and pattern of brainwaves tells us how much energy the brain is using during the operation and can detect abnormalities. Recording them will not harm your child.

The aims of this study are to:

1. Record EEG during, and around the time of aortic surgery.
2. Observe the amount and type of brainwaves before, during and after the operation.
3. Assess whether EEG can improve patient care.

During the operation, your child will be cooled to a core body temperature of 18°C (AKA deep hypothermia) to protect the brain from working too hard. EEG can tell us the optimal temperature to protect your child's brain and we hope to use it in the future to guide the depth of hypothermia. EEG can also help the clinical team who look after your child after the surgery (detailed in **study benefits** below). This study could provide the basis for a much larger study, the results of which could lead to EEG being used in more hospitals which may improve patient care.

What are we asking you to do, and what will your child go through if he or she takes part?

You do not have to do anything after giving consent for your child to take part. In addition to the standard operation, your child will have an EEG recorded before, during, and for 24 hours after their surgery. The doctors/research team will go through the information sheet with you and give you the opportunity to ask any questions. If you are happy for your child to be included in the study, a member of the research team will ask your permission to:

1. Access data on your child's medical and surgical history* and with your permission, your child's GP will be informed of their participation.
2. Apply and analyse the EEG (described in detail below).
3. Contact you 24-months after the surgery to see if you would like us to assess your child's development.

*This information will include your child's initials, gender, hospital number, name, and contact details. People will use this information to do the research or to check your child's records to make sure that the research is being done properly. We need to manage your child's records in specific ways for the research to be reliable. This means that we won't be able to let you see or change the data we hold for them.

Electroencephalography (EEG)

As part of the research study we will place between 15-23 electrodes on your child, which are like sticky pads put on the head and skin (Please refer to *figure one* for image). The electrodes record naturally occurring brain waves – they are just like an ECG tracing of the heart but instead for the brain. They cause no harm and have no side effects but rarely the stickers may cause some redness on the skin when we take them off. EEG will not affect your child's surgery but if there are any unexpected findings, we will let the clinical team know.

Why should I take part and what are the potential benefits of taking part?

Results of the 24-month appointment will be given to your child's doctor. Taking part could improve the care of future children with similar conditions. EEG detects abnormal brainwaves which can cause seizures, and others which indicate brain damage. If we see such things, we will

inform your child's clinical team. The reason we want to do this study is because we think EEG monitoring might improve patient care but there is not enough evidence to support this yet.

What if something goes wrong?

The standard care of children undergoing heart surgery involves intensive monitoring and we do not expect the study itself to cause any problems. Complications of surgery can occur and these will be dealt with in the normal manner, regardless of the research study. Your child's safety during and after surgery is paramount. In the unlikely event that any harm should occur as a result of taking part in this study, we want you to be informed of your rights. There are no special compensation arrangements, but you may have the right to claim damages in a court of law; this would require you to prove fault on the part of the NHS Trust, University or any manufacturer involved. The standard NHS complaints mechanisms are available to you; further information can be obtained from the Patient Advice & Liaison Service (PALS) at Birmingham Children's Hospital on 0121 333 8611.

Does your child have to take part?

Taking part in the study is entirely voluntary – you decide. This parent information sheet gives you information about the study and we can answer any questions that you may have after reading it. Before your child's surgery, one of the research team will ask you whether you wish your child to participate in this study and if so, to sign a consent form. Your child will only be included in the study if you give your express permission. Your child can stop being part of the study at any time, without you giving a reason, but we will keep information about them that we already have. Their surgery will proceed as planned, without any additional measurements, and it will not affect the standard of care that your child receives.

Will my taking part in the study be kept confidential?

Yes. People who do not need to know who your child is will not be able to see their name or contact details. Your child's data will have a code number instead. We will keep all information about your child safe and secure. Paper copies of their data will be stored in a locked office at the hospital. The information from the study will be analysed, presented at scientific meetings,

and published in medical journals to inform other doctors and health professionals of the research findings. All necessary measures will be taken to keep your child's data safe and to comply with the Data Protection Act. EEG and clinical data may be shared with other researchers in the UK or elsewhere, but this will be fully anonymised, such that the other researchers will not be able to link it to your child. Following completion of the study, the data will be kept for 10 years then destroyed in accordance with national guidelines, including any data shared with other researchers. You can find out more about how we use your information at www.hra.nhs.uk/information-about-patients, or by asking a member of the research team.

What happens at the end of the study?

At the end of the study your child's treatment and follow-up continues as would that of a child who had not been involved in the study. Once we have finished the study, we will keep some of the data so we can check the results and contact you in the future. We will write our reports in a way that no-one can work out that your child took part in the study.

Are there any disadvantages to taking part in the study?

No. All tests are safe and your child's routine clinical care will not be affected unless we detect an EEG abnormality.

How will I find out about the results of the study?

The results will be written into a study report after we have analysed all the data. We will send you a newsletter with the results of the study.

Who is organising, funding, and sponsoring this study?

This study has been organised and developed by the teams at Birmingham Children's Hospital. It is funded by the National Institute for Health and Care Research (NIHR) and sponsored by Birmingham Women's and Children's NHS Foundation Trust.

Who has reviewed this research study and leaflet?

The study has been reviewed by the Research & Development team at Birmingham Children's Hospital. It has been given a favourable opinion for conduct in the NHS by the Sheffield Research Ethics Committee. This Parent Information Sheet has been reviewed and revised by the parents of children who have had heart surgery, through the local children's charity *Young at Heart*.

Questions? Contact Mr William McDevitt, Clinical Scientist and Principal investigator for this study by email: w.mcdevitt@nhs.net or via phone on 0121 333 9260

Flow chart for the SAFE Monitoring study

You will have been given this information sheet by a member of the research/clinical team on the ward.

↓

Please read it carefully and consider whether you would like your child to take part. If you have any questions, please feel free to ask.

↓

If you are happy for your child to take part, you will be asked to sign a Consent form by a member of the research team.

↓

On the day of surgery, a member of the research team will apply the EEG electrodes. You can then go along to theatre with your child as usual.

↓

The operation will go ahead as normal and will not be influenced by the EEG.

↓

After the operation, they will be transferred to the Paediatric Intensive Care Unit (PICU) as usual, and you will be able to see them there.

↓

Whilst they are recovering on PICU, the EEG will record data for 24 hours.

↓

When they are well enough, your child will be transferred to the ward and then discharged from hospital once they are ready to go home.

↓

After discharge, they will be seen regularly in the outpatient clinic. We will contact you via telephone to see if you would like to attend an in-hospital assessment that lasts 90 minutes.

↓

Once the study has completed, we can send you a report with the results.

Thank you for taking the time to read this information sheet.
