# Supplementary material for: Surgery on the aortic arch and feasibility of electroencephalography (SAFE) monitoring in neonates: protocol for a prospective observational cohort study
Source: BMJ Open. 2025 Jul 10;15(7):e106423. doi: 10.1136/bmjopen-2025-106423 (PMC12258354; doi:10.1136/bmjopen-2025-106423)
Supplement: online supplemental file 3 [file bmjopen-15-7-s003.pdf]

Birmingham Women's and Children's
NHS Foundation Trust

PARENT/GUARDIAN CONSENT FORM

Surgery on the Aortic arch and Feasibility of EEG Monitoring (SAFE Monitoring).

Chief investigator: Prof. Stefano Seri, Consultant in Clinical Neurophysiology, BCH.

Please initial all boxes

1. I confirm that I have read and understand the patient information sheet (4.0, 04/06/25) for the above study. I have had the opportunity to consider the information, ask questions and have had these answered satisfactorily and am happy for my child to participate.

2. I understand that my child's participation is voluntary and that I am free to withdraw at any time without giving reason, without my child's medical care or legal rights being affected.

3. I understand that relevant sections of my child's medical records and data collected during the study may be looked at by individuals from regulatory authorities or from the NHS trust where it is relevant to my child's taking part in this research. I give permission for these individuals to have access to my child's data.

4. I consent to the storage, including electronic, of personal information for the purposes of this study. I understand that any information that could identify me or my child will be kept strictly confidential, and that no personal information will be included in the study report or other publication.

5. I agree to my child's GP and/or other doctors involved in their care being informed of my child's participation in the study.

6. I understand that the information collected from my child will be used to support other research in the future and may be shared anonymously with other researchers.

7. I consent to being contacted in the future for my child's development to be assessed.

Name of Child: Date of birth:

Name of Parent/Guardian	Signature of Parent/Guardian	Date signed
Name of Investigator	Signature of Investigator	Date signed

Once completed: 1 for parent(s), 1 for medical notes and 1 (original) for trial site file
